# Ten Simple Rules for the Care and Feeding of Scientific Data

**DOI:** 10.1371/journal.pcbi.1003542

**Published:** 2014-04-24

**Authors:** Alyssa Goodman, Alberto Pepe, Alexander W. Blocker, Christine L. Borgman, Kyle Cranmer, Merce Crosas, Rosanne Di Stefano, Yolanda Gil, Paul Groth, Margaret Hedstrom, David W. Hogg, Vinay Kashyap, Ashish Mahabal, Aneta Siemiginowska, Aleksandra Slavkovic

**Affiliations:** 1 Harvard University, Cambridge, Massachusetts, United States of America; 2 University of California, Los Angeles, Los Angeles, California, United States of America; 3 New York University, New York, New York, United States of America; 4 University of Southern California, Los Angeles, Los Angeles, California, United States of America; 5 Vrije Universiteit Amsterdam, Amsterdam, The Netherlands; 6 University of Michigan, Ann Arbor, Michigan, United States of America; 7 California Institute of Technology, Pasadena, California, United States of America; 8 Pennsylvania State University, State College, Pennsylvania, United States of America; University of California San Diego, United States of America

## Introduction

**Figure 1:**
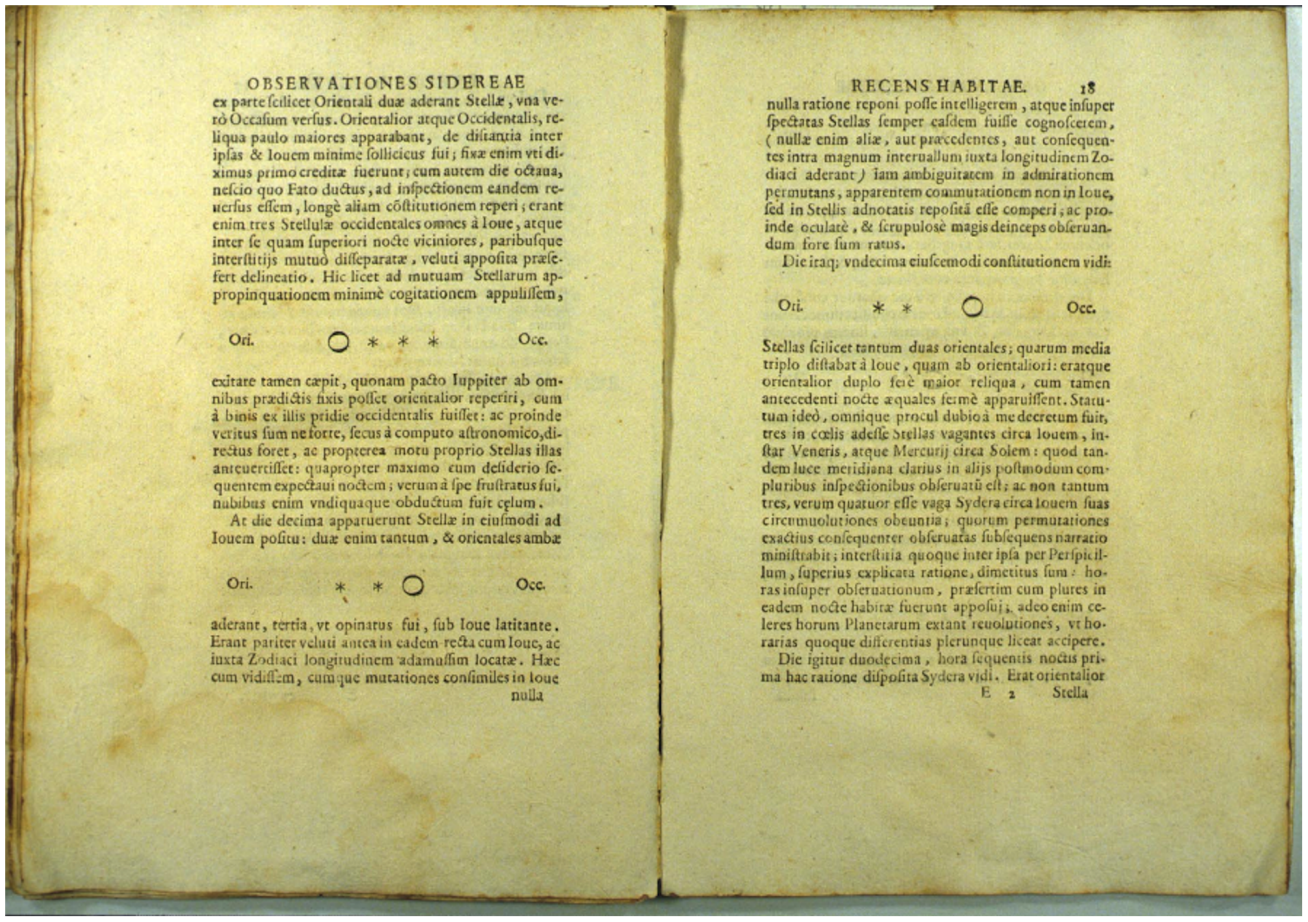
Two pages (scan) from Galilei's *Sidereus Nuncius* (“The Starry Messenger” or “The Herald of the Stars”), Venice, 1610. On these pages, Galilei combines data (drawings of Jupiter and its moons), key metadata (timing of each observation, weather, and telescope properties), and text (descriptions of methods, analysis, and conclusions).

In the early 1600s, Galileo Galilei turned a telescope toward Jupiter. In his log book each night, he drew to-scale schematic diagrams of Jupiter and some oddly moving points of light near it. Galileo labeled each drawing with the date. Eventually he used his observations to conclude that the Earth orbits the Sun, just as the four Galilean moons orbit Jupiter. History shows Galileo to be much more than an astronomical hero, though. His clear and careful record keeping and publication style not only let Galileo understand the solar system, they continue to let *anyone* understand *how* Galileo did it. Galileo's notes directly integrated his **data** (drawings of Jupiter and its moons), key **metadata** (timing of each observation, weather, and telescope properties), and **text** (descriptions of methods, analysis, and conclusions). Critically, when Galileo included the information from those notes in *Sidereus Nuncius* [1], this integration of text, data, and metadata was preserved, as shown in Figure 1. Galileo's work advanced the “Scientific Revolution,” and his approach to observation and analysis contributed significantly to the shaping of today's modern “scientific method” [2], .

Today, most research projects are considered complete when a journal article based on the analysis has been written and published. The trouble is, unlike Galileo's report in *Sidereus Nuncius*, the amount of real data and data description in modern publications is almost never sufficient to repeat or even statistically verify a study being presented. Worse, researchers wishing to build upon and extend work presented in the literature often have trouble recovering data associated with an article after it has been published. More often than scientists would like to admit, they cannot even recover the data associated with their own published works.

Complicating the modern situation, the words “data” and “analysis” have a wider variety of definitions today than at the time of Galileo. Theoretical investigations can create large “data” sets through simulations (e.g., The Millennium Simulation Project: http://www.mpa-garching.mpg.de/galform/virgo/millennium/). Large-scale data collection often takes place as a community-wide effort (e.g., The Human Genome project: http://www.genome.gov/10001772), which leads to gigantic online “databases” (organized collections of data). Computers are so essential in simulations, and in the processing of experimental and observational data, that it is also often hard to draw a dividing line between “data” and “analysis” (or “code”) when discussing the care and feeding of “data.” Sometimes, a copy of the code used to create or process data is so essential to the use of those data that the code should almost be thought of as part of the “metadata” description of the data. Other times, the code used in a scientific study is more separable from the data, but even then, many preservation and sharing principles apply to code just as well as they do to data.

So how do we go about caring for and feeding data? Extra work, no doubt, is associated with nurturing your data, but care up front will save time and increase insight later. Even though a growing number of researchers, especially in large collaborations, know that conducting research with sharing and reuse in mind is essential, it still requires a paradigm shift. Most people are still motivated by piling up publications and by getting to the next one as soon as possible. But, the more we scientists find ourselves wishing we had access to extant but now unfindable data [4], the more we will realize why bad data management is bad for science. How can we improve?

**This article offers a short guide to the steps scientists can take to ensure that their data and associated analyses continue to be of value and to be recognized.** In just the past few years, hundreds of scholarly papers and reports have been written on questions of data sharing, data provenance, research reproducibility, licensing, attribution, privacy, and more—but our goal here is *not* to review that literature. Instead, we present a short guide intended for researchers who want to know why it is important to “care for and feed” data, with some practical advice on how to do that. The final section at the close of this work (Links to Useful Resources) offers links to the types of services referred to throughout the text. **Boldface lettering** below highlights actions one can take to follow the suggested rules.

## Rule 1. Love Your Data, and Help Others Love It, Too

Data management is a repeat-play game. If you take care to make your data easily available to others, others are more likely to do the same—eventually. While we wait for this new sharing equilibrium to be reached, you can take two important actions. First, cherish, document, and **publish your data**, preferably using the robust methods described in Rule 2. Get started now, as better tools and resources for data management are becoming more numerous, universities and research communities are moving toward bigger investments in data repositories (Rule 8), and more librarians and scientists are learning data management skills (Rule 10). At the very least, loving your own available data will serve *you*: you'll be able to find and reuse your own data if you treat them well. Second, enable and **encourage others to cherish, document, and publish their data**. If you are a research scientist, chances are that not only are you an author, but also a reviewer for a specialized journal or conference venue. As a reviewer, **request that the authors of papers you review provide documentation and access to their data** according to the rules set out in the remainder of this article. While institutional approaches are clearly essential (Rules 8 and 10), changing minds one scientist at a time is effective as well.

## Rule 2. Share Your Data Online, with a Permanent Identifier

Nothing really lasts forever, so “permanent” actually just means long-lasting. For example, your personal web site is unlikely to be a good option for long-term data storage (yet, in the very short run, putting your data on your site is better than doing nothing at all!). In general, although many papers include URLs to give access to datasets, most become inaccessible within a few years [5]. The best option for releasing your data with long-term guarantee is to **deposit them in whatever data archive is the “go to” place for your field**. A proper, trustworthy archive will: (1) assign an identifier such as a “handle” (hdl) or “digital object identifier” (doi); (2) require that you provide adequate documentation and metadata; and (3) manage the “care and feeding” of your data by employing good curation practices. If no such archive exists in your field, there are also generic (non-domain-specific) online services that can host your data and issue persistent identifiers (see Rule 8). Pointers to a few generic repositories are listed in the Links to Useful Resources (section A), and longer compilations of such services are in the Links to Useful Resources (B).

## Rule 3. Conduct Science with a Particular Level of Reuse in Mind

Data from others are hard to use without context describing what the data are and how they were obtained. The W3C Provenance Group (http://www.w3.org/TR/2013/REC-prov-dm-20130430/#dfn-provenance) defines information “provenance” as the sum of all of the processes, people (institutions or agents), and documents (data included!) that were involved in generating or otherwise influencing or delivering a piece of information. Perfect documentation of provenance is rarely, if ever, attained in scientific work today. The higher the quality of provenance information, the higher the chance of enabling data reuse. In general, data reuse is most possible when: 1) data; 2) metadata (information describing the data); and 3) information about the process of generating those data, such as code, are all provided. In trying to follow the rules listed in this article, you will do best if you plan in advance for ways to provide all three kinds of information. **In carrying out your work, consider what level of reuse you realistically expect and plan accordingly.** Do you want your work to be fully *reproducible*? If so, then provenance information is a must (e.g., working pipeline analysis code, a platform to run it on, and verifiable versions of the data). Or do you just want your work to be *inspectable*? If so, then intermediate data products and pseudo-code may be sufficient. Or maybe your goal is that your data is *usable* in a wide range of applications? If so, **consider adopting standard formats and metadata standards early on**. At the very least, **keep careful track of versions of data and code**, with associated dates. Taking these steps as you plan and carry out projects will earn you the thanks of researchers, including you, looking back from the future. (Consult the Links to Useful Resources [E] for a list of tools to package all your research materials with reuse in mind.)

## Rule 4. Publish Workflow as Context

Publishing a description of your processing steps offers essential context for interpreting and reusing data. As such, scientists typically include a “methods” and/or “analysis” section(s) in a scholarly article, used to describe data collection, manipulation, and analysis processes. Computer and information scientists call the combination of the collection methods and analysis processes for a project its “workflow,” and they consider the information used and captured in the workflow to be part of the “provenance” of the data. In some cases (mostly in genomics), scientists can use existing workflow software in *running* experiments and in *recording* what was done in those experiments, e.g., Gene Pattern (www.genepattern.org). In that best-case scenario, the workflow software, its version, and settings used can be published alongside data using the other rules laid out here. But, it is rare outside of genomics to see the end-to-end process described in a research paper run, orchestrated, and/or recorded by a single software package. In a plausible utopian future, automated workflow documentation could extend to all fields, so that an electronic provenance record could link together all the pieces that led to a result: the data citation (Rule 2), the pointer to the code (Rule 6), the workflow (this rule), and a scholarly paper (Rule 5). But what can you do now? **At a minimum, provide, alongside any deposit of data, a simple sketch of data flow across software, indicating how intermediate and final data products and results are generated. If it's feasible and you are willing to deal with a higher level of complexity, also consider using an online service to encapsulate your workflow (see** **Links to Useful Resources** **[C] for a list of services)**. Keep in mind that even if the data used are not “new,” in that they come from a well-documented archive, it is still important to document the archive query that produced the data you used, along with all the operations you performed on the data after they were retrieved. Keeping better track of workflow, as context, will likely benefit you and your collaborators enough to justify the loftier, more altruistic, goals espoused here.

## Rule 5. Link Your Data to Your Publications as Often as Possible

Whether your “data” include tables, spreadsheets, images, graphs, databases, and/or code, you should make as much of it as possible available *with* any paper that presents it. **If it's practical and helpful, share your data as early as possible in your research workflow: as soon as you are done with the analysis, even before you write any articles about it.** Your data can even be cited before (or without) its inclusion in a paper (see Rule 7). Many journals now offer standard ways to contribute data to their archives and link it to your paper, often with a persistent identifier. Whenever possible, **embed citations (links) to your data and code, each with its own persistent identifier, right into the text of your paper, just like you would reference other literature.** If a journal hosting your paper doesn't offer a place for your data, and or an identifier for it, use a repository (Rule 8) and get your own identifier (Rule 2). At a *minimum*, you can post, and refer to, a package of files (data, codes, documentation on parameters, metadata, license information, and/or lists of links to such) with a persistent online identifier (Rule 2). And, if your domain's journals' policies do not allow for good data–literature interlinking, try to effect change (see Rules 1 and 10).

## Rule 6. Publish Your Code (Even the Small Bits)

Did you write any code to run your analysis? **No matter how buggy and insignificant you may find it, publish it.** Many easy-to-use source code repositories exist, which allow not only hosting of software but also facilitate collaboration and version tracking (see Links to Useful Resources [D]). Your code, even the shortest script (whether or not you are proud of its quality), can be an important component for understanding your data and how you got your results [6]. Software plays several roles in relation to data and scientific research, and norms around its publication are still evolving and differ across disciplines [7]. In some cases, software is the primary data product (e.g., new algorithms). In some other cases, data are the primary research products, yet the best way to document their provenance is to publish the software that was used to generate them as “metadata.” In both cases, publishing the source code and its version history is crucial to enhance transparency and reproducibility. The use of open-source software when possible reduces barriers for subsequent users of your software-related data products [8]. The same best practices discussed above in relation to data and workflow also apply to software materials: cite the software that you use and provide unique, persistent identifiers (Rule 2) to the code you share.

## Rule 7. State How You Want to Get Credit

Chances are that you want to get credit for what you share. The attribution system used for scholarly articles, accomplished via citations, often breaks in the case of data and software. When other authors reuse or cite your data or code, you may get an acknowledgment or an incoming link. If you and your colleagues have gone to the trouble to write a “data paper,” whose main purpose is to describe your data and/or code, you may also get a citation [9]. But, “data paper” writing is not always desirable, or relevant. So, how do you go about getting the full credit you deserve for your data and code? **The best way is to simply describe your expectations on how you would like to be acknowledged.** If you want, **you can also release your data under a license and indicate explicitly in the paper or in the metadata how you want others to give you credit.** But, while legal mechanisms have advantages, they can also inadvertently lead to limitations on the reuse of the data you are sharing. In any case, **make information about you (e.g., your name, institution), about the data and/or code (e.g., origin, version, associated files, and metadata), and about exactly how you would like to get credit, as clear as possible.** Easy-to-implement licenses, many of which offer the advantage of being machine-readable, are offered by the Creative Commons organization (http://creativecommons.org/choose/), as are other similar options, such as those offered by Open Data Commons (http://opendatacommons.org/licenses/pddl/). The Links to Useful Resources (G) provides more information.

## Rule 8. Foster and Use Data Repositories

Sometimes the hardest and most time-consuming step of sharing data and code is finding and deciding where to put them. Data-sharing practices vary widely across disciplines: in some fields data sharing and reuse are essential and commonplace, while in others data sharing is a “gift exchange” culture [10]. **If your community already has a standard repository, use it. If you don't know where to start looking, or you need help choosing among relevant repositories, ask an information specialist, such as a data scientist or a librarian working in your field** (and consult the directories of data repositories listed in the Links to Useful Resources [B]). When choosing among repositories, try to find the one offering the best combination of ease-of-deposit, community uptake, accessibility, discoverability, value-added curation, preservation infrastructure, organizational persistence, and support for the data formats and standards you use. **Remember that even if your field has no domain-based repository, your institution may have one**, and your local librarian or archivist can instruct you on how to use that local resource. If neither your community nor your institution has a relevant repository, try a generic repository or consider setting up your own (see Rule 2, and Links to Useful Resources [F]).

## Rule 9. Reward Colleagues Who Share Their Data Properly

Whether you do it in person at scientific meetings and conferences or by written communication when reviewing papers and grants, **reward your colleagues who share data and code. Rally your colleagues and engage your community by providing feedback on the quality of the data assets in your field. Praise those following the best practices.** The more the data created by your colleagues is accessible as an organized collection of some sort, the better your community's research capacity. The more data get shared, used, and cited, the more they improve. Besides personal involvement and encouragement, the best way to reward data sharing is by attribution: always cite the sources of data that you use. **Follow good scientific practice and give credit to those whose data you use, following their preferred reference format and according to current best practices.** Standards and practices for citing and attributing data sources are actively being developed through international partnerships [11], [12].

## Rule 10. Be a Booster for Data Science

As Rule 1 says, it is important not just that *you* love your own data, but that *others* love data, too. An attitude that data and code are “second-class objects,” behind traditional scholarly publications, is still prevalent. But, every day, as scientists try to use the frustrating but tantalizing hodgepodge of research data available via the present ad hoc network of online systems, the value of organizing an open network of reusable data and code is becoming more and more clear, to more and more people. **You, as a scientist, need to help organize your discipline and your institution to move more quickly toward a world of open, discoverable, reproducible data and research. One important step is to** ***advocate*** **for hiring data specialists and for the overall support of institutional programs that improve data sharing.** Make sure not only advanced researchers (e.g., postdocs) experience the pleasures of doing research with freely available data and tools: ***explain*** **and** ***show*** **the value of well-loved data to graduate and undergraduate researchers**. *Teach* whole courses, or mini-courses, related to caring for data and software, or incorporate the ideas into existing courses. *Form groups* specific to your discipline to foster data and code sharing. Hold birds-of-a-feather or special *sessions during large meetings* demonstrating examples in which good sharing practices have led to better results and collaborations. Lead by practicing what you preach.

## Links to Useful Resources

### A: General Data Repositories

- Dataverse (http://thedata.org): A repository for research data that takes care of long-term preservation and good archival practices, while researchers can share, keep control of, and get recognition for their data.
- FigShare (http://figshare.com): A repository where users can make all of their research outputs available in a citable, shareable, and discoverable manner.
- Zenodo (http://zenodo.org): A repository service that enables researchers, scientists, projects, and institutions to share and showcase multidisciplinary research results (data and publications) that are not part of existing institutional or subject-based repositories.
- Dryad (http://datadryad.org): A repository that aims to make data archiving as simple and as rewarding as possible through a suite of services not necessarily provided by publishers or institutional websites.

### B: Directories of Research Data Repositories

- DataBib (http://databib.org): Databib is a tool for helping people identify and locate online repositories of research data. Users and bibliographers create and curate records that describe data repositories that users can search.
- Re3data.org (http://www.re3data.org): Re3data is a global registry of research data repositories from different academic disciplines for researchers, funding bodies, publishers, and scholarly institutions.
- Open Access Directory (http://oad. simmons.edu/oadwiki/Data_repositories): A list of repositories and databases for open data.
- Force 11 Catalog (http://www.force11.org/catalog): A dynamic inventory of web-based scholarly resources, a collection of alternative publication systems, databases, organizations and groups, software, services, standards, formats, and training tools.

### C: Workflow Management Systems

- Taverna (http://www.taverna.org.uk): An open-source and domain-independent workflow management system—a suite of tools used to design and execute scientific workflows and aid in silico experimentation.
- Kepler (https://kepler-project.org): Software designed to help scientists, analysts, and computer programmers create, execute, and share models and analyses across a broad range of scientific and engineering disciplines.
- Wings (http://www.wings-workflows.org): A semantic workflow system that assists scientists with the design of computational experiments.
- VisTrails (http://www.vistrails.org): An open-source scientific workflow and provenance management system that supports data exploration and visualization.
- Knime (http://www.knime.org): A graphical workbench for the entire analysis process: data access, data transformation, initial investigation, powerful predictive analytics, visualization, and reporting.

### D: Source Code Repositories

- Github (http://github.com): A web-based hosting service for software development projects that use the Git revision control system, including many open-source projects.
- Git (http://git-scm.com): A free and open-source distributed version control system designed to handle everything from small to very large projects with speed and efficiency.
- Mercurial (http://mercurial.selenic.com): A free, distributed source control management tool. It efficiently handles projects of any size and offers an easy and intuitive interface.
- BitBucket (https://bitbucket.org): A web-based hosting service for projects that use either the Mercurial or Git revision control systems.

### E: Systems to Package, Access, and Execute Data and Code

- IPython Notebook (http://ipython.org/notebook.html): A web-based interactive computational environment where you can combine code execution, text, mathematics, plots, and rich media into a single document.
- ROpenSci (http://ropensci.org): A suite of packages that allow access to data repositories through the R statistical programming environment.
- Authorea (https://authorea.com): A collaborative online word processor for scholarly papers that allows the writing of web-native, living, dynamic, “executable” articles that include text, mathematical notation, images, and data. It currently supports inclusion and rendering of d3.js plots and IPython notebooks.
- Dexy (http://dexy.it): A multipurpose project automation tool for working with documents via a command-line interface.

### F: Software Tools to Run Your Own Document Repository

- Invenio (http://invenio-software.org): Invenio is a free software suite enabling you to run your own digital library or document repository on the web. Invenio is an ideal solution for running document repositories of moderate to large sizes (several millions of records). Invenio is codeveloped by CERN, DESY, EPFL, FNAL, and SLAC.
- Eprints (http://www.eprints.org/software): EPrints is one of the easiest and fastest ways to set up small to medium-sized repositories of open-access research literature, scientific data, theses, reports, and multimedia. Developed at the University of Southampton, UK.
- DSpace (http://www.dspace.org): DSpace is a turnkey institutional repository application developed by the Duraspace organization.

### G: Licensing and Privacy

- Open Source Initiative (http://opensource.org/licenses): Open-source licenses are licenses that comply with the Open Source Definition: they allow software to be freely used, modified, and shared. These include Apache, BSD, GNU (GPL), MIT, and the Mozilla Public License.
- Privacy Tools for Sharing Research Data (http://privacytools.seas.harvard.edu): A Harvard-based collaborative and multidisciplinary effort to help enable the collection, analysis, and sharing of personal data for research in social science and other fields while providing privacy for individual subjects.
